# Longitudinal analysis of anthropometric measures over 5 years in patients with Friedreich ataxia in the EFACTS natural history study

**DOI:** 10.1111/ene.70011

**Published:** 2025-01-11

**Authors:** Stella Andrea Lischewski, Kerstin Konrad, Imis Dogan, Claire Didszun, Ana Sofia Costa, Sara Annabelle Schawohl, Paola Giunti, Michael H. Parkinson, Caterina Mariotti, Lorenzo Nanetti, Alexandra Durr, Claire Ewenczyk, Sylvia Boesch, Wolfgang Nachbauer, Thomas Klopstock, Claudia Stendel, Francisco Javier Rodríguez de Rivera Garrido, Ludger Schöls, Zofia Fleszar, Thomas Klockgether, Marcus Grobe‐Einsler, Ilaria Giordano, Myriam Rai, Massimo Pandolfo, Jörg B. Schulz, Kathrin Reetz, the EFACTS study group, Elisabetta Indelicato, Matthias Ampros, Cinzia Gellera, Alessia Mongelli, Anna Castaldo, Mario Fichera, Enrico Bertini, Gessica Vasco, Marie Biet, Marie Lorraine Monin, Florian Holtbernd, Nikolina Brcina, Christian Hohenfeld, Florentine Radelfahr, Almut T. Bischoff, Stefanie N Hayer, Georgios Koutsis, Marianthi Breza, Francesc Palau, Mar O’Callaghan, Gilbert Thomas‐Black, Katarina Manso, Nita Solanky, Robyn Labrum

**Affiliations:** ^1^ Department of Neurology RWTH Aachen University Aachen Germany; ^2^ JARA‐BRAIN Institute Molecular Neuroscience and Neuroimaging, Forschungszentrum Jülich GmbH Jülich Germany; ^3^ Child Neuropsychology Section, Department of Child and Adolescent Psychiatry, Psychosomatics and Psychotherapy University Hospital, RWTH Aachen Germany; ^4^ Ataxia Centre, Department of Clinical and Movement Neurosciences UCL‐Queen Square Institute of Neurology London UK; ^5^ Unit of Medical Genetics and Neurogenetics Fondazione IRCCS Istituto Neurologico Carlo Besta Milan Italy; ^6^ Paris Brain Institute, ICM Institut du Cerveau, AP‐HP, INSERM, CNRS, University Hospital Pitié‐Salpêtrière, Sorbonne Universite Paris France; ^7^ Department of Neurology Medical University Innsbruck Innsbruck Austria; ^8^ Department of Neurology Friedrich Baur Institute, University Hospital, LMU Munich Germany; ^9^ German Center for Neurodegenerative Diseases Munich Germany; ^10^ Munich Cluster for Systems Neurology Munich Germany; ^11^ Reference Unit of Hereditary Ataxias and Paraplegias, Department of Neurology IdiPAZ, Hospital Universitario La Paz Madrid Spain; ^12^ Department of Neurology and Hertie‐Institute for Clinical Brain Research University of Tübingen Tübingen Germany; ^13^ German Center for Neurodegenerative Diseases Tübingen Germany; ^14^ Department of Neurology University Hospital of Bonn Bonn Germany; ^15^ German Center for Neurodegenerative Diseases Bonn Germany; ^16^ Friedreich Ataxia Research Alliance Downingtown Pennsylvania USA; ^17^ Laboratory of Experimental Neurology Université Libre de Bruxelles Brussels Belgium

**Keywords:** body height, body mass index, Friedreich ataxia, natural history, underweight

## Abstract

**Background:**

Friedreich ataxia is a rare neurodegenerative disorder caused by frataxin deficiency. Both underweight and overweight occur in mitochondrial disorders, each with adverse health outcomes. We investigated the longitudinal evolution of anthropometric abnormalities in Friedreich ataxia and the hypothesis that both weight loss and weight gain are associated with faster disease progression.

**Methods:**

Participants were drawn from the European Friedreich's Ataxia Consortium for Translational Studies (EFACTS). Age‐ and sex‐specific BMI and height scores were calculated using the KIGGS‐BMI percentiles for children. Height correction was applied for scoliosis. Longitudinal data were analysed using linear mixed effects models and incremental standard deviation scores and growth mixture models identified subclasses with varying BMI trajectories.

**Results:**

Five hundred and forty‐three adults and fifty‐nine children were assessed for up to 5 years. In children, severe underweight (26%), underweight (7%), severe short stature (16%) and short stature (23%) were common. The corrected BMI percentile was stable in children, although 48% had negative incremental BMI scores over 1 year and 63% over 3 years versus 10%/year in a normal reference cohort. Overweight was common in adults (19%), with a slight increase in BMI over time. Longer GAA repeat size was linked to lower BMI in adults. Weight trajectory was not associated with ataxia progression in adults.

**Conclusion:**

Significant anthropometric abnormalities were identified, with underweight and short stature prevalent in children and overweight in adults. These findings highlight the need for regular nutritional monitoring and interventions to manage underweight in children and promote healthy weight in adults.

## INTRODUCTION

Friedreich ataxia is a rare genetic multisystem neurodegenerative disorder, usually starting around puberty but with variable onset [1]. Most individuals carry a biallelic GAA repeat expansion in the frataxin (*FXN*) gene with repeat expansion length serving as the most important known prognostic factor, explaining approximately 30% of variation in age of onset, onset and time until loss of ambulation [2, 3]. Frataxin plays a key role in multiple mitochondrial processes, including adenosine triphosphate (ATP) production. Therefore, Friedreich ataxia is mainly driven by mitochondrial failure.

Underweight is prevalent in children with mitochondrial disorders [4], including Friedreich ataxia [5], and body mass index (BMI) correlates with ATP production [6]. Underweight may be associated with numerous adverse health outcomes, including delayed puberty [5], loss of lean muscle mass [7], exacerbation of muscle weakness, fatigue and depression. Conversely, adults with Friedreich ataxia are frequently overweight [5], which has been ascribed to reduced mobility with advancing disease progression and exercise intolerance related to mitochondrial dysfunction [5, 8]. Overweight may increase the risk of cardiomyopathy and diabetes [9, 10] and may interfere with engagement with physiotherapy and physical activity, conceivably hastening disease progression.

Previous work on the US‐ and Australian‐based FACOMS register has examined height and the pubertal growth spurt longitudinally, and BMI cross‐sectionally. We sought to extend this analysis to investigate the longitudinal evolution of BMI and to examine the hypothesis that both underweight/weight loss and obesity/weight gain, compared with normal weight/stable weight, are linked to accelerated disease progression.

A better understanding could yield insights to guide further research into the efficacy of targeted nutritional support and aid in monitoring nutritional status in clinical practice.

Our objectives were as follows:

1. To determine the prevalence of anthropometric abnormalities in patients with Friedreich ataxia and to identify factors associated with BMI and height.
2. To explore changes in BMI and height over a 5‐year follow‐up period, including the existence of subgroups with differential evolution of BMI and height, and the association with disease progression, risk of diabetes and cardiomyopathy.

## METHODS

### Standard protocol approvals, registrations and patient consents

This study adheres to the STROBE guidelines for reporting observational studies [11]. Approval was received by the institutional review boards of the respective study centres (EK 057/10, Medizinische Ethik‐Kommission RWTH Aachen University). Written informed consent was received from all participants or legal guardians for underage participants prior to participating in the study. The study conforms to the declaration of Helsinki.

### Participants and anthropometric assessments

Participants were drawn from the European Friedreich's Ataxia Consortium for Translational Studies (EFACTS) registry, an international observational cohort study with currently up to 5 years of follow‐up data. Detailed methods are described elsewhere [3, 12, 13]. Briefly, patients with genetically confirmed Friedreich ataxia were seen annually in 11 centres across Europe. Disease progression was measured using the Scale for the Assessment and Rating of Ataxia (SARA) and the activities for daily living (ADL). Cardiac hypertrophy was defined as diagnosed by the local cardiologist. Body mass index was calculated using the formula: BMI = weight/height [2]. For adults, BMI reference ranges were as defined by the World Health Organization [14]. For children and adolescents below the age of 18 years, age‐ and sex‐specific BMI and height scores were calculated according to the KIGGS‐BMI reference provided by the Robert Koch Institute [15]. Here, the cut‐off values were as follows: severe underweight/short stature <3rd percentile, underweight/short stature 3–10th percentile, normal 10–90th percentile, overweight/tall stature 90–97th percentile and obese/very tall stature >97th percentile. Given that a proportion of our paediatric cohort transitioned into adulthood (>18 years) during the study period and that reference percentiles are not reported for this age group, we performed a sensitivity analysis, including follow‐up data of these participants using percentiles for individuals aged 17 years.

### Height correction for scoliosis

Scoliosis was categorized into absent, mild, moderate or severe according to clinical judgement [1]. This information was used to approximate the Cobb angle, the angle between the lines drawn along the superior endplate of the most tilted vertebra above the curve and the inferior endplate of the most tilted vertebra below the curve. Patients with mild, moderate and severe scoliosis were classified as having Cobb angles of 15, 30 and 45, respectively [16]. Height loss due to scoliosis was subsequently corrected for by using the Bjure formula [17]. Sensitivity analyses were applied where correction for scoliosis was omitted.

### Statistical analysis

The association between factors with BMI and height at baseline was determined using univariate and multivariable linear regression. Change in BMI and height over time were examined using a linear mixed effects model corrected for sex with a random intercept and fixed slope due to difficulties with model convergence when fitting a random slope. We employed an age scale using age as a fixed effect as this was deemed to be the main disease evolution factor for chronic disease in previous literature [18]. To address the positively skewed BMI and height data in children in the linear mixed effects models, a square root transformation was applied to normalize its distribution. The variable importance factor was examined to identify any multicollinearity. Scatter plots were used to identify outliers and to explore the linearity of the relationship; Q‐Q plots were examined to determine the normality of residuals.

As recommended in paediatric literature, we examined annual increments and conditional change in corrected BMI and height standard deviation scores (SDS) to examine growth trends in children in addition to linear mixed effect models [19]. SDS scores denote the increment in corrected BMI or height Z‐score over a specified follow‐up period.

To identify subclasses of adult participants with differential BMI evolution trajectories, we employed a growth mixture model, including latent variables. BMI measurements at each timepoint were used to determine a slope and intercept for each latent class, with no constraints imposed on these parameters. No covariates were included in the model. To determine the optimal number of latent classes, we considered the Bayesian information criterion (BIC), the parametric bootstrap likelihood test and the Vuong‐Lo–Mendell–Rubin test alongside the substantive interpretability of the model and class size [20]. To evaluate classification quality, we reported the entropy measure. Latent class analysis models were fitted using maximum likelihood estimation with robust standard errors. Finally, we examined the relationship between BMI class membership and changes in SARA and ADL scores using a linear mixed effects model. Missing data in latent growth mixture modelling were handled using full information maximum likelihood procedures.

We performed joint modelling to explore whether the time until the first missing BMI measurement was related to BMI. All *p*‐values are two‐tailed. Given the exploratory nature of the study, no adjustments were made for multiple comparisons. Descriptive statistics are presented as mean and standard deviation (SD) or median and interquartile range (IQR). Analyses were performed in MPlus version 8.4 and R version 4.3.1.

## RESULTS

### Baseline characteristics

**FIGURE 1:**
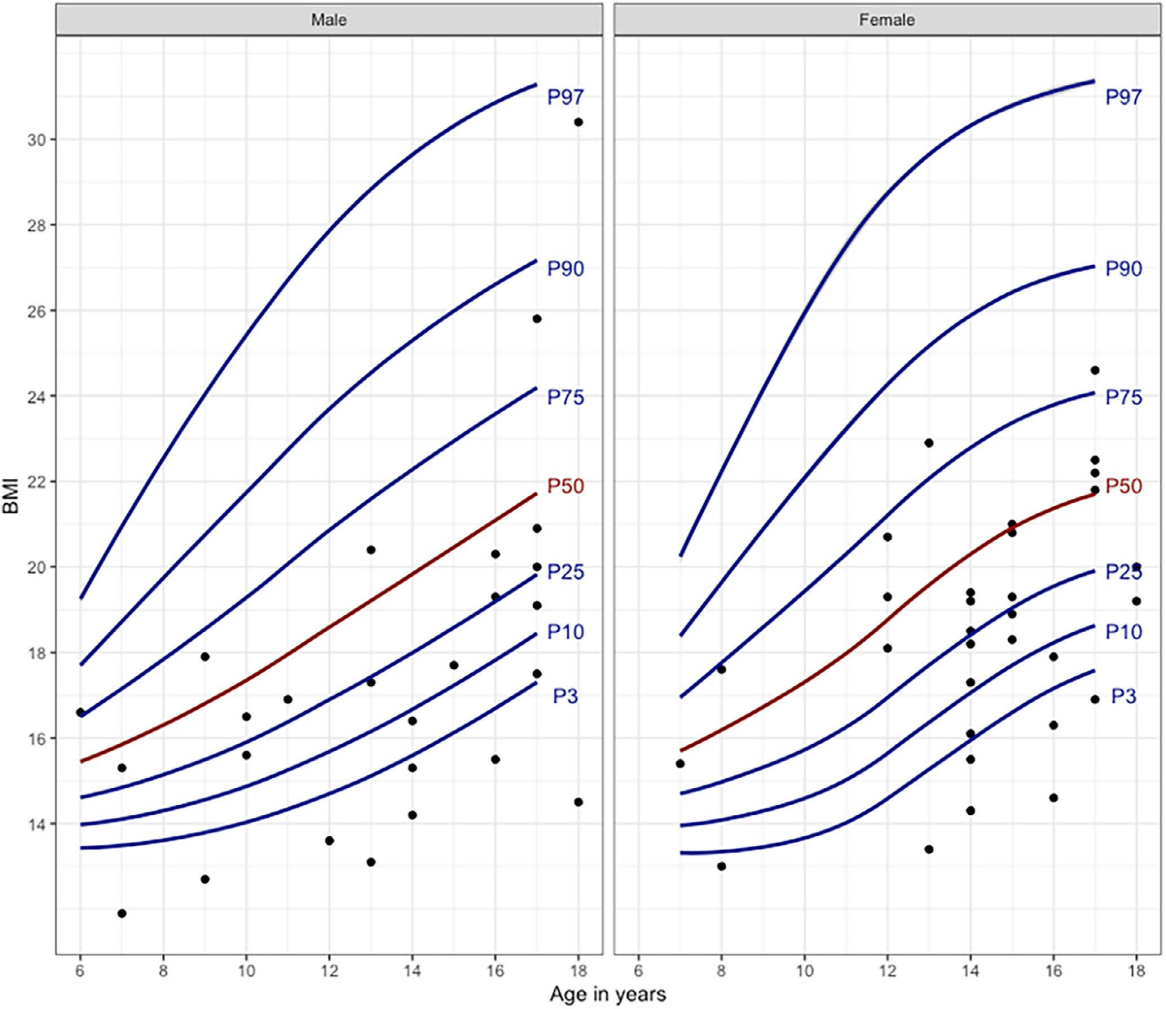
Corrected BMI according to percentiles in male and female children at baseline. Legend: Each dot represents one child. BMI in children was defined as follows: severe underweight <3rd percentile, underweight <3–10th percentile, normal 10–90th percentile, overweight 90–97th percentile and obese >97th percentile. P3, 3rd percentile; P10, 10th percentile; P25, 25th percentile; P50, 50th percentile; P75, 75th percentile; P90, 90th percentile; P97, 97th percentile, BMI, body mass index.

**FIGURE 2:**
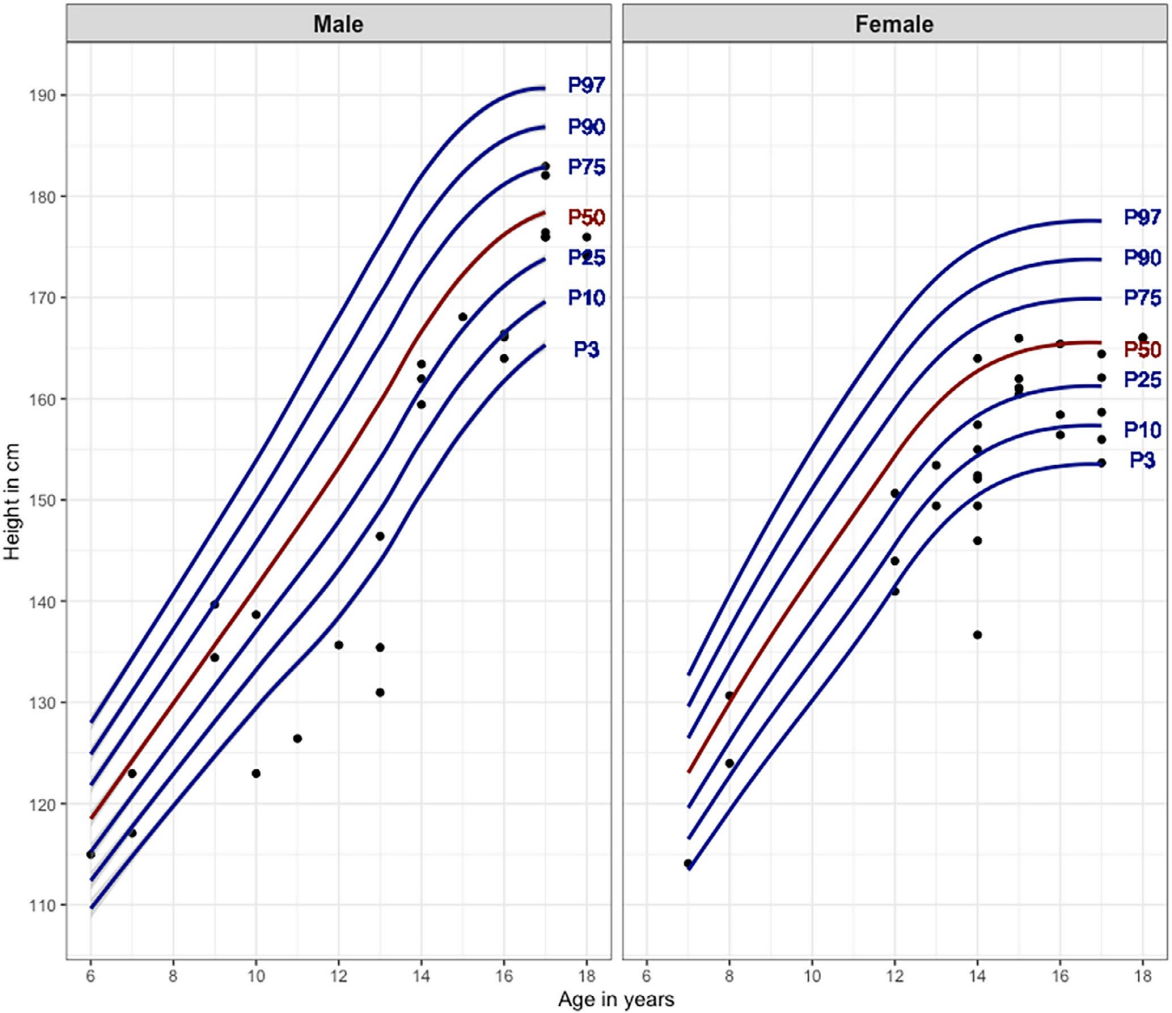
Corrected height according to percentiles in male and female children at baseline. Each dot represents one child. Height in children was defined as follows: severe short stature <3rd percentile, short stature 3–10th percentile, normal 10–90th percentile, tall stature 90–97th percentile and very tall stature >97th percentile. P3, 3rd percentile; P10, 10th percentile; P25, 25th percentile; P50, 50th percentile; P75, 75th percentile; P90, 90th percentile; P97, 97th percentile.

Overall, 602 participants (59 children and 543 adults) were included with a median of 3.8 visits for each participant (Table 1). Severe underweight and underweight were prevalent in children (26% and 7% respectively, Figure 1) but rare in adults (0.8% and 9% respectively). In contrast, overweight was prevalent in adults (19%) but rare in children (1.8%). Severe short stature (< 3rd percentile) and short stature (<10th percentile) were prevalent in children (16% and 23% respectively, Figure 2).

**TABLE 1:** Main demographic and clinical characteristics of children and adults at baseline.

Characteristics	Overall *N* = 602^a^	Children *N* = 59^a^	Adults *N* = 543^a^
Age in years	32 (23, 43)	14 (12, 16)	35 (26, 45)
Sex (female)	324 (54%)	32 (54%)	292 (54%)
Age at symptom onset	13 (9, 19)	7 (4, 9)	14 (10, 20)
Disease duration	17 (10, 25)	6 (4, 8)	18 (12, 26)
GAA repeats shorter allele	650 (385, 800)	780 (680, 850)	616 (367, 783)
BMI category corrected			
Severe underweight	19 (3.4%)	15 (26%)	4 (0.8%)
Underweight	50 (8.9%)	4 (7.0%)	46 (9.1%)
Normal	367 (65%)	37 (65%)	330 (65%)
Overweight	95 (17%)	1 (1.8%)	94 (19%)
Obese	30 (5.3%)	0 (0%)	30 (6.0%)
Corrected height category			
Severe short stature	30 (5.2%)	9 (16%)	21 (4.0%)
Short stature	47 (8.2%)	13 (23%)	34 (6.6%)
Normal	450 (78%)	35 (61%)	415 (80%)
Tall	29 (5.0%)	0 (0%)	29 (5.6%)
Very tall	20 (3.5%)	0 (0%)	20 (3.9%)
SARA total score	23 (13, 31)	12 (9, 21)	24 (15, 31)
ADL total score	14 (8, 20)	7 (4, 14)	15 (9, 21)
Ambulatory	287 (48%)	48 (81%)	239 (44%)
Scoliosis	198 (33%)	22 (37%)	176 (32%)
Cardiac hypertrophy	216 (38%)	30 (55%)	186 (36%)
Spasticity	118 (24%)	7 (13%)	111 (26%)
Muscle atrophy	297 (50%)	24 (41%)	273 (51%)
Gastrointestinal symptoms	79 (13%)	1 (1.7%)	78 (14%)
Dysphagia (ADL)			
0	154 (26%)	37 (63%)	117 (22%)
1	253 (43%)	15 (25%)	238 (44%)
2	180 (30%)	7 (12%)	173 (32%)
3	5 (0.8%)	0 (0%)	5 (0.9%)
4	2 (0.3%)	0 (0%)	2 (0.4%)
Depression	NA	NA	70 (13%)
SSRI intake	39 (6.5%)	0 (0%)	39 (7.2%)

*Note*: BMI categories in adults were defined as follows: severe underweight <16, underweight 16–18.4, normal weight 18.5–24.9, overweight 25–29.9, obesity >30. Height categories in adults are based on the percentile values for a 17‐year old. BMI category and height in children was defined as follows: severe underweight/short stature <3rd percentile, underweight/short stature <3–10th percentile, normal 10–90th percentile, overweight/tall stature 90–97th percentile and obese/very tall stature >97th percentile.

Abbreviations: ADL, activities of daily living; BMI, body mass index; GAA, guanine adenine adenine; SARA, Scale for the Assessment and Rating of Ataxia.

^a^ Median (Q1, Q3); *n* (%).

In adults, male sex, older age at onset, longer disease duration, shorter GAA repeat expansion length on the shorter allele, absence of scoliosis, absence of muscle atrophy and presence of gastrointestinal symptoms were all associated with higher corrected BMI in univariate analyses (Table 2). Similarly, all aforementioned factors except GAA repeat length were associated with BMI in multivariable analyses (Table S1a,b). Interestingly, ambulatory status was not associated with BMI, nor was there an interaction effect between age of onset and ambulatory status on BMI.

**TABLE 2:** Results of univariate regression for demographic and clinical factors with corrected BMI (adults) and corrected BMI percentile (children) at baseline.

Characteristic	Children (*n* = 59)			Adults (*n* = 543)		
	Beta	95% CI	*p*‐value	Beta	95% CI	*p*‐value
Sex (female)	3.8	−11, 18	0.6	−0.93	−1.6, −0.24	0.009
Age at symptom onset	−0.53	−3.0, 1.9	0.7	0.08	0.05, 0.12	<0.001
Disease duration	0.08	−2.2, 2.4	>0.9	0.06	0.03, 0.10	<0.001
GAA repeats shorter allele standardized	−3.4	−12, 5.1	0.4	−0.55	−0.90, −0.20	0.002
ADL total score	0.21	−0.92, 1.3	0.7	−0.02	−0.07, 0.03	0.4
Ambulatory	6.7	−12, 25	0.5	−0.12	−0.82, 0.58	0.7
Scoliosis	−3.6	−11, 4.1	0.4	−1	−1.4, −0.67	<0.001
HbA1c	0.63	−0.38, 1.6	0.2	0.03	−0.03, 0.09	0.3
Cardiac hypertrophy	0.24	−15, 16	>0.9	−0.87	−1.6, −0.11	0.024
Spasticity	5.9	−17, 29	0.6	0.43	−0.41, 1.3	0.3
Muscle atrophy	−5.4	−20, 9.3	0.5	−1.6	−2.3, −0.91	<0.001
Gastrointestinal symptoms	56	2.7, 110	0.04	2.5	1.5, 3.5	<0.001
Dysphagia (ADL)	−11	−21, −0.59	0.038	−0.13	−0.59, 0.34	0.6
Depression	NA	NA	NA	−0.13	−2.4, 2.1	>0.9
SSRI intake	NA	NA	NA	1.1	−0.24, 2.5	0.11

*Note*: Univariate linear regression with listed characteristics as respective independent variables and corrected BMI (adults) or corrected BMI percentile (children) as the dependant variable.

Abbreviations: ADL, activities of daily living; CI, confidence interval.

In children, only presence of dysphagia was associated with lower BMI in univariate analysis (Table 2) and no factors were significant in multivariable analyses (Table S1a,b). No factors were associated with height in children in univariate or multivariable analyses (Tables S2 and S3).

### Availability of follow‐up data for BMI

The proportion of missing data for weight and height increased over time (baseline weight: 7%, height 4%, visit 5 weight 13% and height 5%). Missingness was higher in non‐ambulatory patients (baseline weight: 9% non‐ambulatory vs. 2% ambulatory; height: 6% ambulatory vs. 2% non‐ambulatory, visit 5: weight 5% ambulatory vs. 18% non‐ambulatory and height: 6% ambulatory vs. 2% non‐ambulatory). Missingness was also higher in adults than in children (baseline weight: 6% adults vs. 3% children, height: 4% adults vs. 3% children and visit 5: weight 14% adults vs. 3% children and height 5% adults vs. 0% children). Time until first missing BMI measurement was not related to BMI.

### Rate of change in BMI, BMI percentile and height percentile over time

**FIGURE 3:**
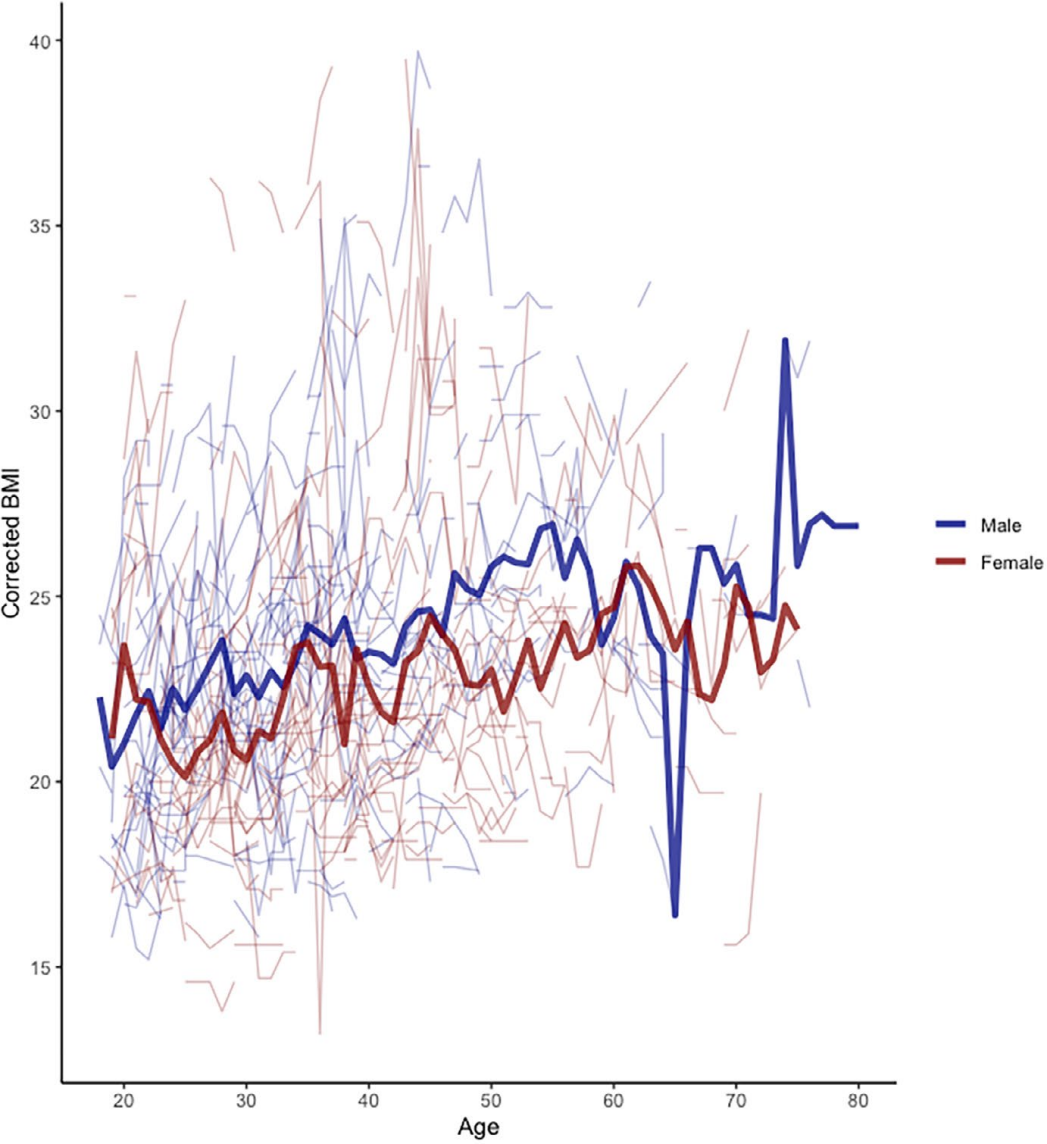
Corrected BMI according over time in male and female adults. Mean corrected BMI over time in men and women. BMI: body mass index.

In adults, BMI increased slightly over time (0.09 kg/m^2^ per year ±0.011 (SD) [95% CIs 0.065, 0.11], *p* < 0.001, Figure 3); an analysis using visit rather than age and an analysis omitting the scoliosis correction yielded similar results (data not shown). Women had a lower BMI than men (−1.1 kg/m^2^ ± 0.33 (SD) [95% CIs −0.44, −1.8], *p* < 0.001, Figure 3). There was no interaction between time (age) and sex (data not shown). A subgroup analysis of patients with late onset (over 25 years) yielded a stable BMI over time (0.02 kg/m^2^ per year ±0.028 (SD) [95% CIs −0.02, 0.08], *p* < 0.4, Figure S1). Height did not change substantially over time (Figure S2).

In children, the square root‐transformed corrected BMI percentile was stable over time (−0.1 per year ±0.08 (SD) [95% CIs −0.25, 0.06]; *p* = 0.24) (Figure S3). Over 1 year, 15/31 (48%) children had negative incremental BMI SDS scores; this number increased to 16/23 (70%) over 2 years, 12/19 (63%) over 3 years and 9/14 (63%) over 4 years compared with 10% per year in a normal reference cohort [19].

**FIGURE 4:**
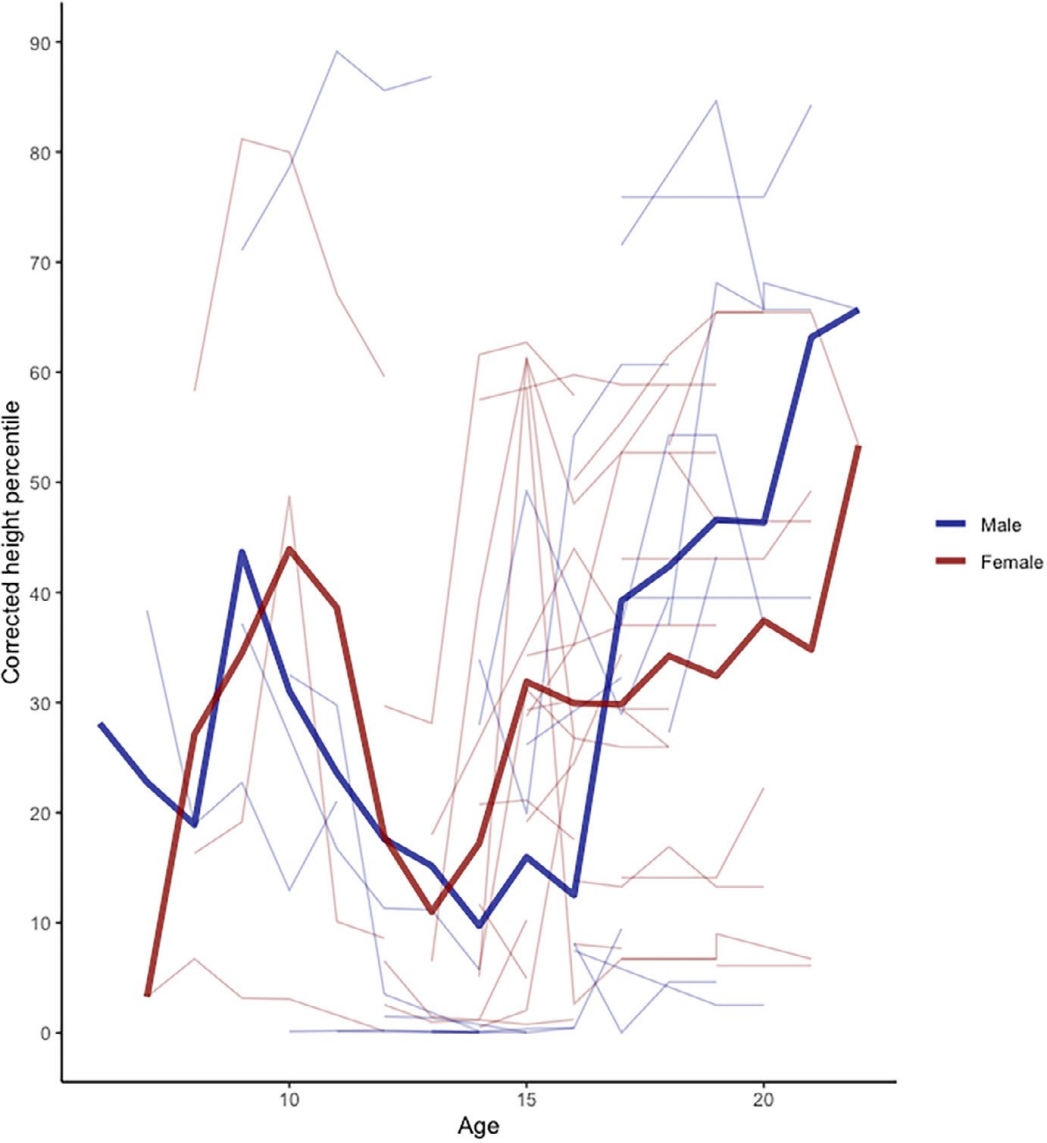
Corrected height percentile in male and female children over time. Mean corrected height percentile in male and female children.

The square root‐transformed corrected height percentile also remained stable over time (0.073 ± 0.074 (SD) [95% CIs −0.07, 0.22]; *p* = 0.32). Graphically, height percentile peaked at around age 10, decreased during early puberty and recovered after approximately age 15 (Figure 4). The nadir and subsequent recovery occurred slightly later in boys than in girls.

Here, 14/31(45%) of children had negative incremental height SDS scores over 1 year whilst this number dropped to 9/23 (39%) over 2 years, 7/19 (36%) over 3 years and 8/14 (57%) over 4 years. There was no difference in BMI (0.84 ± 0.75 (SD) [95% CIs −0.63, 2.3]; *p* = 0.27) or height (0.36 ± 0.66 (SD) [95% CIs −0.93, 1.6]; *p* = 0.59) between girls and boys and no interaction between time (age) and sex.

Sensitivity analyses in children omitting scoliosis correction yielded similar results (data not shown). An analysis, including follow‐up data of participants turning 18 years during the follow‐up duration, showed a stable square root‐transformed corrected BMI percentile (0.2 per year ±0.055 (SD) [95% CIs −0.089, 0.12]; *p* = 0.74) and a smaller number of participants with negative increments in BMI SDS scores (1 year: 20/43 [47%], 2 years: 22/39 [56%], 3 years: 15/34 [44%] and 4 years: 18/35 [51%]). There was a borderline significant increase in square root‐transformed corrected height percentile over time (0.11 ± 0.046 (SD) [95% CIs 0.018, 0.20]; *p* = 0.020). A similar number of participants had negative incremental height SDS scores (1 year: 16/43 (37%), 2 years: 12/39 (31%), 3 years: 13/36 (36%) and 4 years: 11/36 (31%)).

### Trajectories of BMI evolution

Growth mixture models with two to five classes on BMI evolution were fitted in the adult patient population but not in children due to the small sample size. The four‐class model yielded the most meaningful interpretation and was supported by multiple fit indices (Table S4). The four‐class model yielded a class with relatively stable BMI evolution that remained within the normal range (*n* = 467), a class with increasing BMI (*n* = 15), a very small class with decreasing BMI (*n* = 4) and a class with stable but obese BMI (*n* = 39) (Figure S4). We then related class membership to change in SARA and ADL score, respectively, and did not identify a relevant association (Table S5). Members of Class 4 were older with later onset and shorter GAA repeat length; otherwise, there were no substantial differences between latent classes (Table S6).

**FIGURE 5:**
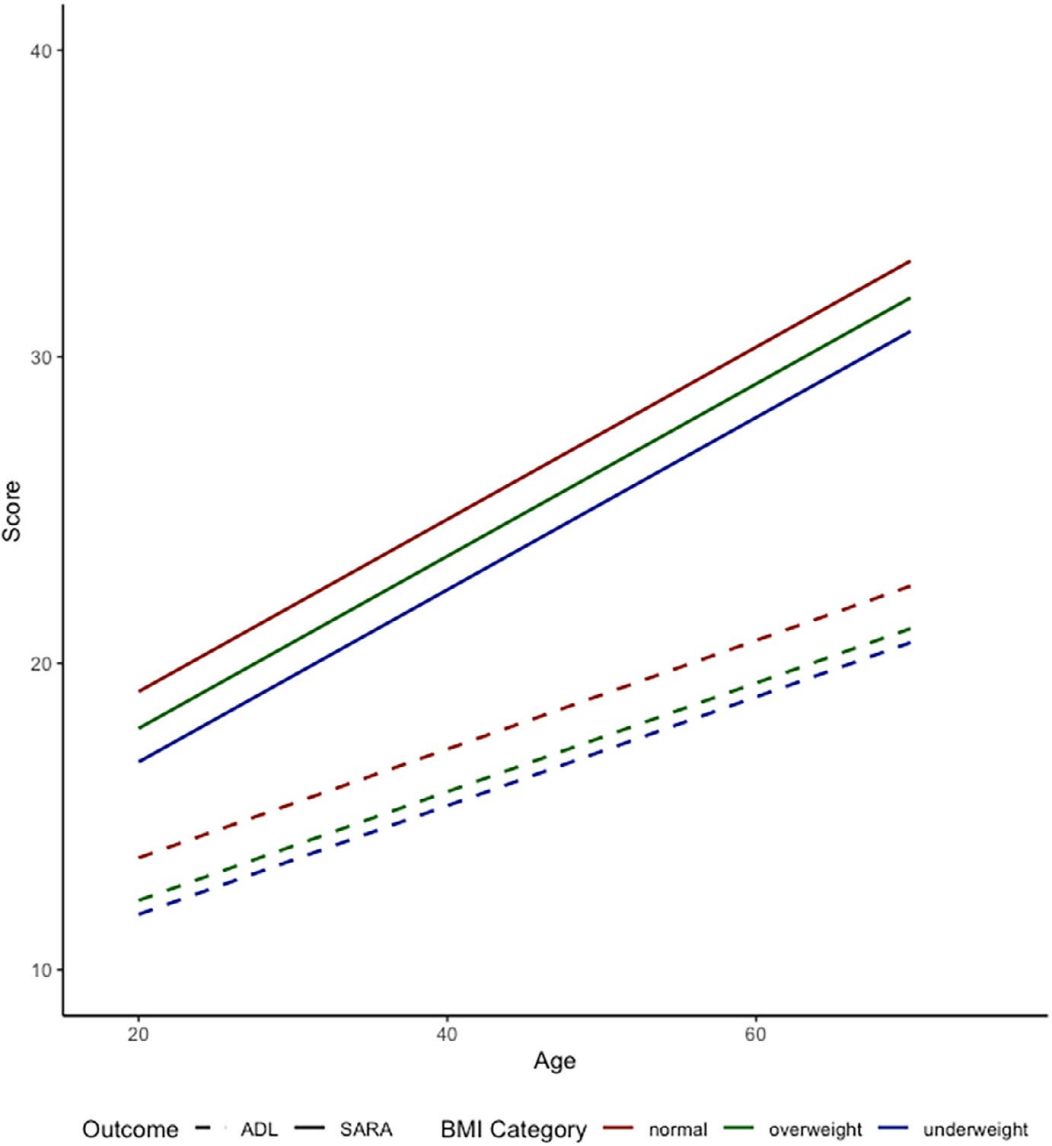
Longitudinal evolution of SARA and ADL score according to corrected BMI category at baseline in adults. Linear mixed effect model with random intercept and random slope showing longitudinal evolution of SARA and ADL score with age in adults according to corrected BMI category. BMI category was defined as follows: Underweight: BMI < 18.5 (*n* = 40), normal: BMI 18.5–24.9 (*n* = 1623), overweight BMI > 25 (*n* = 136). ADL, activities of daily living; BMI, body mass index; SARA, Scale for the Assessment and Rating of Ataxia.

We also compared SARA and ADL score evolution based on classification into overweight, underweight and normal weight at baseline as the reference category, because missingness of BMI data was lowest at baseline but increased over time. After correction for GAA repeat length and disease duration, underweight (−2.1 ± 0.42 [95% CIs −1.3, −2.9]; *p* < 0.001) and, to a lesser degree, overweight (−0.13 ± 0.26, [95% CIs −0.85, −1.8]; *p* < 0.001) were associated with lower SARA scores. Similarly, overweight (−1.4 ± 0.24 [−1.9, −0.9]; *p* < 0.001) and underweight (−1.9 ± 0.49 [−2.8, −0.89]; *p* < 0.001) were associated with lower ADL scores (Figure 5; Table S7 for unadjusted analyses). However, the rate of ataxia progression did not differ to those with normal weight.

### Association of weight category with diabetes and cardiomyopathy

Being overweight at baseline was not associated with HbA1c (−2.0 ± 4.3 (SD) [95% CIs −10.4, 6.5]; *p* = 0.65) or with cardiomyopathy (−0.72 ± 0.41 (SD) [95% CIs −1.5, 0.11]; *p* = 0.086), and neither was underweight (data not shown). The analysis was corrected for age and sex (Table S7 for unadjusted analyses).

## DISCUSSION

In this large prospective European multicentre cohort study, we investigated the prevalence and longitudinal evolution of anthropometric measures in patients with Friedreich ataxia and their impact on disease progression. Overall, underweight and short stature were common in children and a substantial proportion of children dropped their percentile score over time. Conversely, in adults, overweight was prevalent, and BMI increased slightly over time. Underweight and overweight at baseline were associated with lower SARA and ADL score, but there was no relevant difference in the rate of progression.

The prevalence of underweight in children in our European cohort surpassed those in two recent US‐based studies on the FACOMS registry (26% <3rd percentile in our cohort vs. 17% <5th percentile in Ref. [5] and 20% <5th percentile in Ref. [21] compared with 1%–2% in the general European population [22]), perhaps owing to differences in dietary habits. Similarly, 16% had severe short stature, compared with 2% in a healthy British reference population [23]. Additionally, the nadir in height percentile during puberty likely reflects pubertal delay, as height percentile drops when peers enter puberty and recovers as children with Friedreich ataxia catch up, in keeping with previous literature [5].

Whilst the decrease in corrected BMI percentile in the linear mixed effects model was not statistically significant, possibly due to limited sample size, approximately 48% of our paediatric patients had a drop in percentile score over 1 year, compared with 10% in a healthy reference cohort [19]. A likely explanation is that mitochondrial dysfunction is linked to enteral malabsorption, insufficient nutrient utilization and diminished appetite [6]. Additionally, diet alterations were observed in children with mitochondriopathies [24]. Malnutrition in turn was found to cause secondary mitochondrial dysfunction in patients with malignancies [25, 26] and could potentially exacerbate mitochondriopathy in Friedreich ataxia. Additionally, lean muscle mass was noted to be lower in patients with Friedreich ataxia compared with healthy controls [27]. Recent evidence underlines the potential benefits of targeted nutritional support to ameliorate ATP production in children with mitochondriopathies [28] and should be considered in children with Friedreich ataxia at risk of underweight and failure to thrive.

Unlike children, adults with Friedreich ataxia were frequently overweight, consistent with findings from the US‐based cohort (19% compared with 33% in FACOMS [5] and 50% in the European general population aged 35–44 years [29]). However, these results contrast findings on other neurodegenerative disorders, such as spinocerebellar ataxia [18], Huntington disease [30] and amyotrophic lateral sclerosis [31], where weight decline is common and associated with faster disease progression [18, 30, 31]. We also did not identify an association between BMI and ambulatory status. Therefore, it appears unlikely that obesity in adults with Friedreich ataxia is solely due to ataxia‐related immobility and attendant reduction in physical activity, although this may represent a contributing factor. An alternative explanation is offered by a recent experimental study, which identified mitochondrial dysfunction in white adipose cells as a putative mechanism for weight gain in a *FXN* knockout mouse model. Frataxin deficiency in white adipose cells was hypothesized to cause expansion of adipocyte size, hypovascularization, production of pro‐inflammatory adipokines, immune cell recruitment and fibrosis, ultimately leading to weight gain [32]. In this context, an obesogenic diet was found to aggravate the metabolic dysfunction caused by frataxin deficiency in mice [33]. Interestingly, in another mouse model characterized by a comparatively early disease onset and longer GAA repeat length, weight loss rather than weight gain was noted [7]. This finding is corroborated by our result on the high prevalence of underweight in children and overweight in adults as well as our observation that longer GAA repeat length was associated with lower BMI in adults.

Contrary to our initial hypothesis, we found that BMI trajectory was not associated with disease progression in adults with Friedreich ataxia. Underweight and, to a lesser degree, overweight at baseline were associated with a lower SARA and ADL score but there was no difference in rate of disease progression. The cause of this remains unclear, but it is plausible that thinner individuals had higher levels of physical fitness, which in turn had a beneficial effect on SARA score. The association between overweight and lower SARA score may have occurred due to data missing not at random. Our initial hypothesis, based on observations linking obesity to the accelerated onset of cardiomyopathy and diabetes mellitus [9, 10], as well as its likely adverse impact on disease‐related inactivity and effectiveness of physiotherapy [34, 35] was not supported.

We also did not observe an association between BMI and diabetes or cardiomyopathy, which may be because diabetes and pancreatic β‐cell dysfunction is mainly driven by frataxin deficiency rather than cardiovascular risk factors in individuals with Friedreich ataxia [10]. Instead, this lack of association may be linked to the key role of GAA repeat length in mediating BMI. Additional possible explanations include a ceiling effect in the SARA score [36] and the gradual and highly variable nature of Friedreich ataxia; as such, an even more extended follow‐up period may be required to detect significant effects. Lastly, in view of the multisystem nature of Friedreich Ataxia, additional non‐neurological outcomes should be considered in future research.

The strengths of this study include its large sample size and long follow‐up duration of up to 5 years. Furthermore, we employed height correction in patients with scoliosis, a common comorbidity [37]. Notwithstanding these strengths, there are important limitations. These include a substantial proportion of missing data on weight, especially in non‐ambulatory patients, which likely relates to the challenges in obtaining weight measurements in this patient group. In some patients, weight and height may have been estimated rather than determined using weighing scales. Missing or estimated weight data could have resulted in an underestimate of the extent of the BMI increase over time in adult patients or masked an association between BMI and disease progression. To address this, we performed an analysis of SARA evolution based on BMI category at baseline when data completeness was high. Whilst underweight and, to a lesser degree, overweight at baseline was associated with a lower SARA score, there was no difference in rate of disease progression. Given the relatively small number of children in our cohort, the analysis on the effect of BMI on disease progression was not possible; furthermore, the analysis of factors associated with BMI at baseline was likely underpowered. The lack of exact birthdates for our participants, requiring age to be rounded, introduces potential inaccuracies in age‐dependent analyses and could impact the precision of our findings. Depression was assessed using a binary score, more granular screening instruments would have been preferable. Lastly, scoliosis severity, as determined by clinician judgement, is potentially inaccurate. However, sensitivity analyses excluding scoliosis correction yielded similar results.

## CONCLUSION

The disparity between underweight and risk of failure to thrive in children versus overweight and weight gain in adults with Friedreich ataxia aligns with findings in a mouse model and appears to be at least partially mediated by GAA repeat expansion size. After correcting for this, we found no association between BMI trajectory and disease progression although underweight and, to a lesser degree, overweight at baseline was associated with a lower SARA and ADL score. Our study highlights the importance of continued research into the complex metabolic alterations associated with Friedreich ataxia and lays the foundation for further studies on the efficacy of targeted nutritional support. Moreover, our results underscore the importance of a comprehensive care approach for individuals with Friedreich ataxia, emphasizing the necessity for ongoing monitoring of nutritional and physical status. To achieve accurate weight measurements, particularly in non‐ambulatory patients, staff training is paramount. Early interventions to address underweight in children and promote healthy weight management in adults are crucial. In particular, regular strength and coordination training as well as aerobic exercise should be encouraged.

## AUTHOR CONTRIBUTIONS

**Stella Andrea Lischewski:** Conceptualization; methodology; investigation; validation; formal analysis; visualization; writing – original draft; writing – review and editing. **Kerstin Konrad:** Conceptualization; methodology; writing – review and editing; investigation. **Imis Dogan:** Conceptualization; methodology; investigation; writing – review and editing. **Claire Didszun:** Conceptualization; methodology; investigation; writing – review and editing; data curation. **Ana Sofia Costa:** Conceptualization; methodology; investigation; writing – review and editing. **Sara Annabelle Schawohl:** Conceptualization; methodology; investigation; writing – review and editing. **Paola Giunti:** Project administration; funding acquisition; resources; writing – review and editing; supervision. **Michael H. Parkinson:** Project administration; supervision; writing – review and editing. **Caterina Mariotti:** Funding acquisition; project administration; supervision; resources; writing – review and editing. **Lorenzo Nanetti:** Project administration; supervision; resources; writing – review and editing. **Alexandra Durr:** Writing – review and editing; project administration; resources; supervision; funding acquisition. **Claire Ewenczyk:** Project administration; supervision; resources; writing – review and editing. **Sylvia Boesch:** Funding acquisition; project administration; supervision; resources; writing – review and editing. **Wolfgang Nachbauer:** Writing – review and editing; project administration; supervision; resources. **Thomas Klopstock:** Writing – review and editing; project administration; supervision; resources; funding acquisition. **Claudia Stendel:** Writing – review and editing; project administration; resources; supervision. **Francisco Javier Rodríguez de Rivera Garrido:** Funding acquisition; supervision; resources; writing – review and editing; project administration. **Ludger Schöls:** Funding acquisition; writing – review and editing; supervision; resources; project administration. **Zofia Fleszar:** Project administration; supervision; resources; writing – review and editing. **Thomas Klockgether:** Funding acquisition; project administration; supervision; resources; writing – review and editing. **Marcus Grobe‐Einsler:** Project administration; resources; supervision; writing – review and editing. **Ilaria Giordano:** Project administration; supervision; resources; writing – review and editing. **Myriam Rai:** Writing – review and editing; supervision; resources; project administration. **Massimo Pandolfo:** Funding acquisition; project administration; supervision; resources; writing – review and editing. **Jörg B. Schulz:** Funding acquisition; project administration; supervision; resources; writing – review and editing. **Kathrin Reetz:** Conceptualization; methodology; investigation; supervision; resources; project administration; funding acquisition; writing – review and editing.

## FUNDING INFORMATION

Most authors are part of EFACTS, which was funded by an FP7 grant from the European Commission (HEALTH‐F2–2010‐242,193), EuroAtaxia, Voyager Therapeutics and the Christina Foundation. This work was also funded by a grant from the Interdisciplinary Centre of Clinical Research (IZKF) within the faculty of medicine in the Uniklinik RWTH Aachen (OC2).

The funders had no role in the concept and design of the study, the analysis and interpretation of data or the writing and review of the manuscript.

## CONFLICT OF INTEREST STATEMENT

S.A. Lischewski has received speaker honoraria from Biogen. W. Nachbauer has received speaker and advisory honoraria from Biogen and Reata Pharmaceuticals. S. Boesch reports consultancies from VICO Therapeutics, Reata pharmaceuticals and Biogen, honoria from Ipsen, Merz, Abbvie and Reata and advisory boards for Biogen and Reata. L. Schöls received consultancies from VICO Therapeutics, Vigil Neuroscience and Novartis unrelated to this work. K. Konrad, C. Didszun, A. S. Costa, I. Dogan, S. A. Schawohl, P. Giunti, M. H. Parkinson, C. Mariotti, A. Durr, L. Nanetti, T. Klopstock, C. Ewenczyk, C. Stendel, F. J. Rodríguez de Rivera Garrido, Z. Fleszar, T. Klockgether, M. Grobe‐Einsler, I. Giordano, M. Rai, M. Pandolfo, J. B. Schulz and K. Reetz report no disclosures relevant to the manuscript.

## Supporting information


**Data S1:** Supporting Information.

## Data Availability

Researchers who provide a methodologically sound proposal can request individual de‐identified data for meta‐analyses from the EFACTS steering committee by contacting ataxiestudien@ukaachen.de. The data that support the findings of this study are available on request from the corresponding author. The data are not publicly available due to privacy or ethical restrictions.
