# Supplementary material for: Longitudinal analysis of anthropometric measures over 5 years in patients with Friedreich ataxia in the EFACTS natural history study
Source: Eur J Neurol. 2025 Jan 11;32(1):e70011. doi: 10.1111/ene.70011 (PMC11724196; doi:10.1111/ene.70011)
Supplement: Supplementary file 1 — Data S1: Supporting Information. [file ENE-32-e70011-s001.docx]

**Supplementary material:**

**Supplementary Table 1a.** Results of multivariable regression for demographic and clinical factors with corrected BMI (adults) and corrected BMI percentile (children) at baseline

**
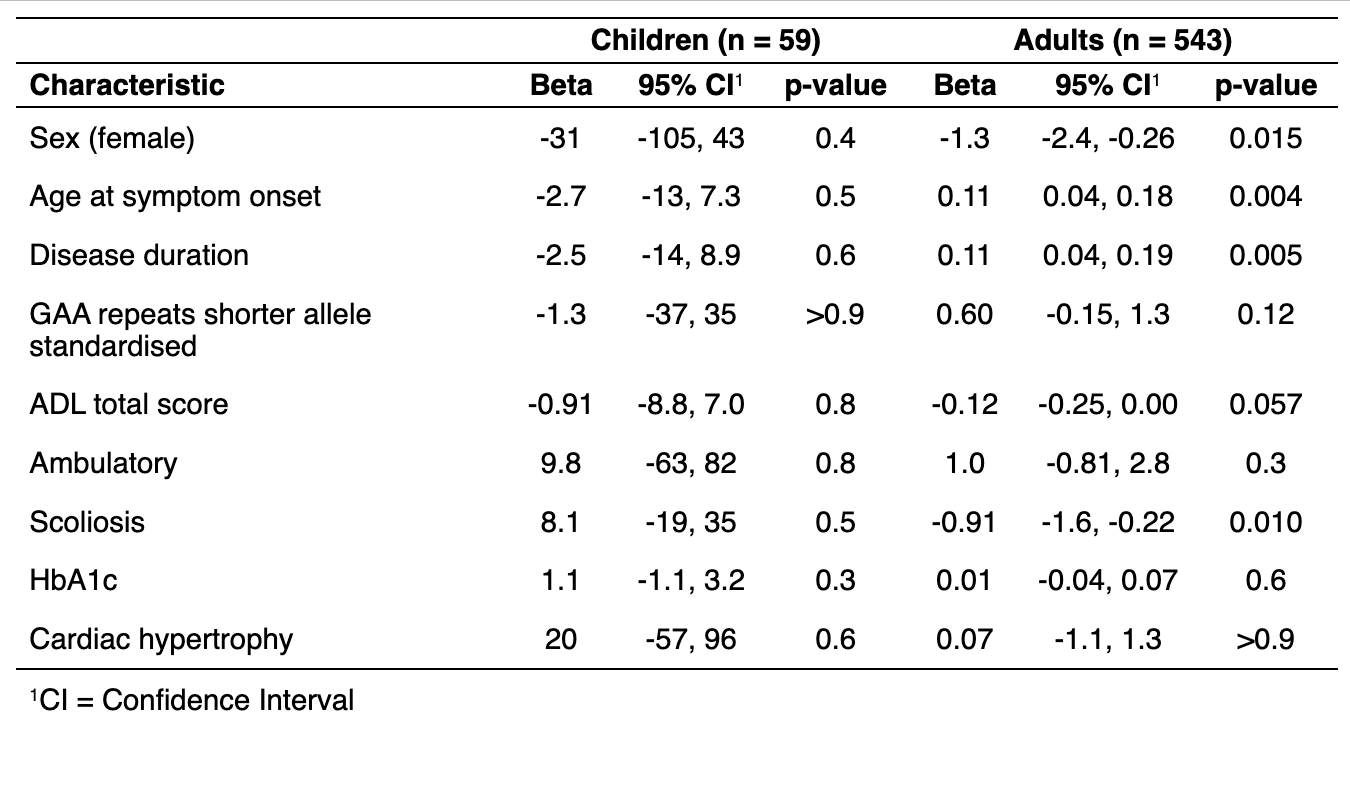
**

**Supplementary Table 1b.** Results of multivariable regression for demographic and clinical factors with corrected BMI (adults) and corrected BMI percentile (children) at baseline


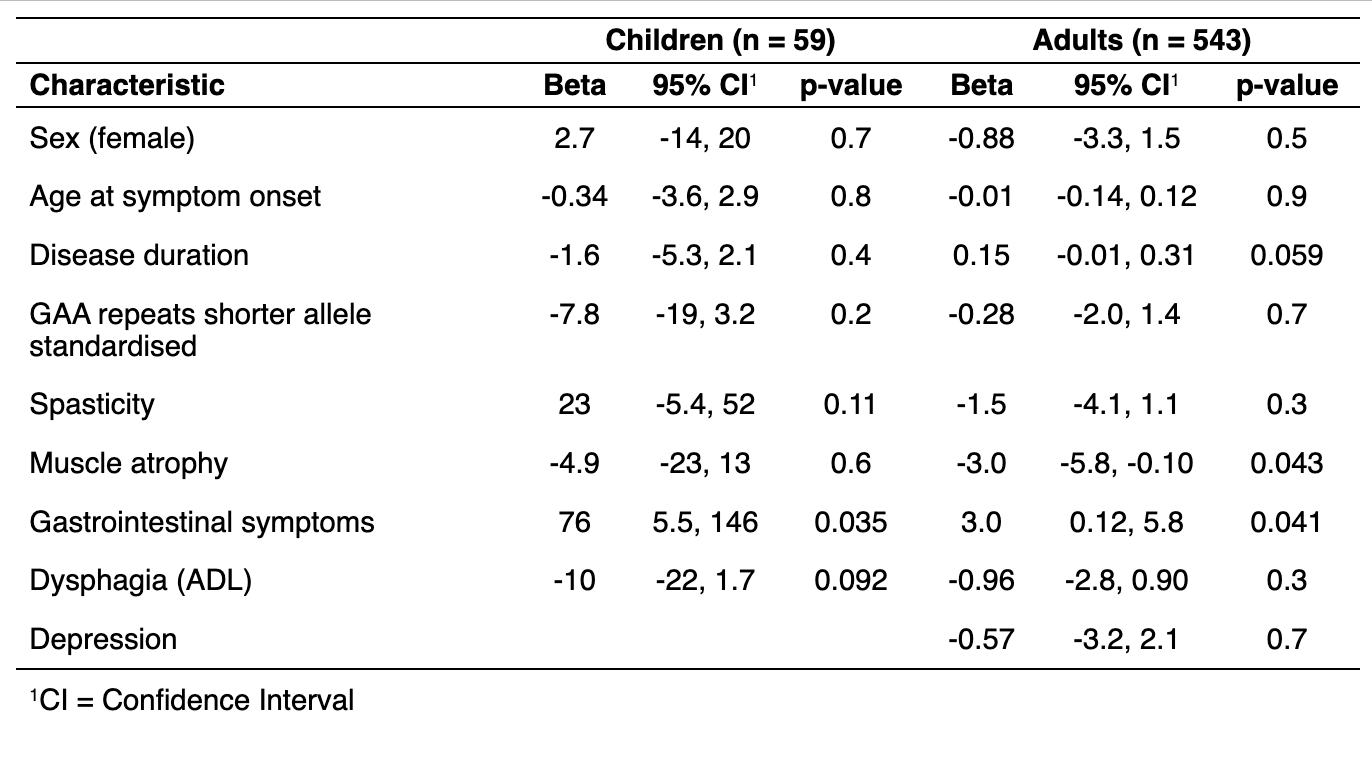


*Multivariable linear regression with listed characteristics as independent variables and corrected BMI (adults) or corrected BMI percentile (children) as the dependant variable. Abbreviations: ADL: activities of daily living. GAA: guanine adenine adenine*

**Supplementary Table 2.** Results of univariate regression for demographic and clinical factors with corrected height percentile in children at baseline


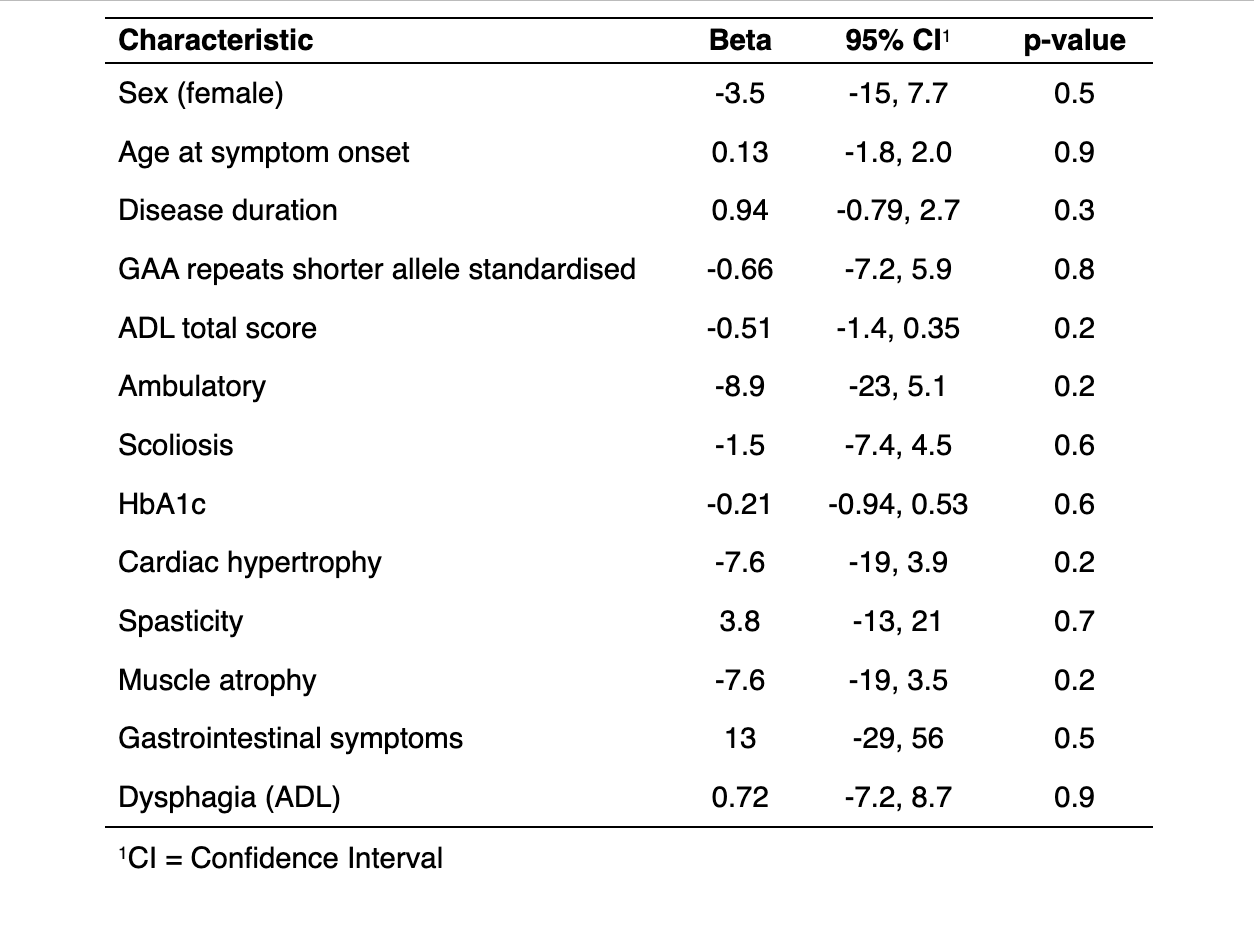


*Univariate linear regression with listed characteristics as independent variables and corrected height percentile as the dependant variable in children. Abbreviations: ADL: activities of daily living. GAA: guanine adenine adenine*

**Supplementary Table 3.** Results of multivariable regression for demographic and clinical factors with corrected height percentile in children at baseline


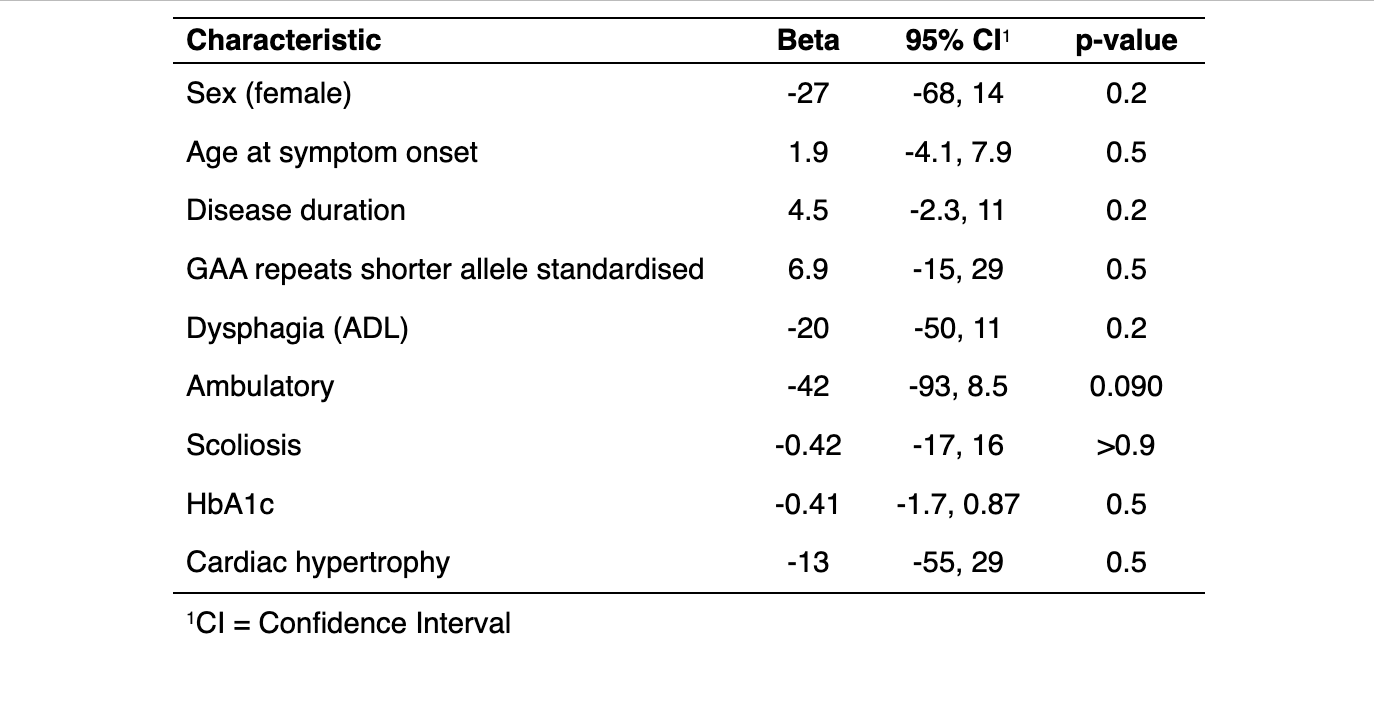


*Multivariable linear regression with listed characteristics as independent variables and corrected height percentile in children as the dependant variable. Abbreviations: ADL: activities of daily living.*

**Supplementary Figure 1.** Longitudinal BMI evolution according to age of onset


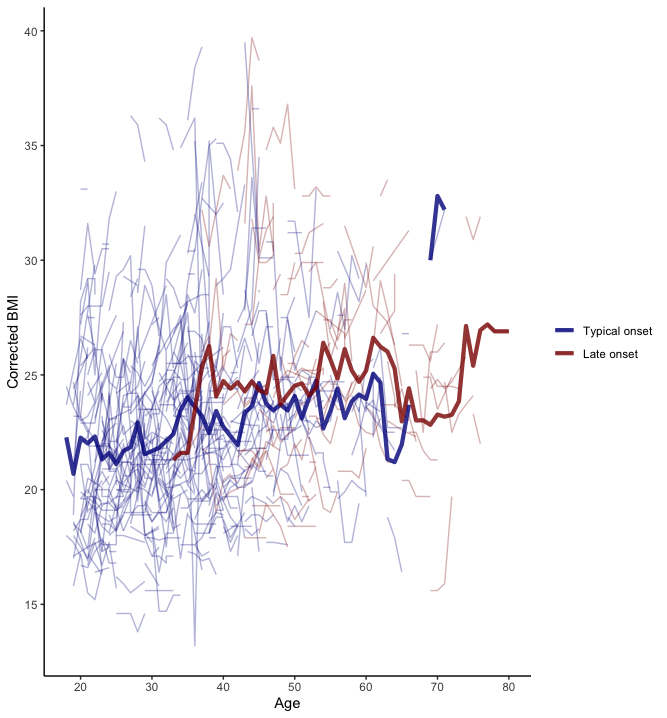


*Mean corrected BMI over time in adults with typical vs late onset.*

**Supplementary Figure 2.** Longitudinal height evolution in adults

**
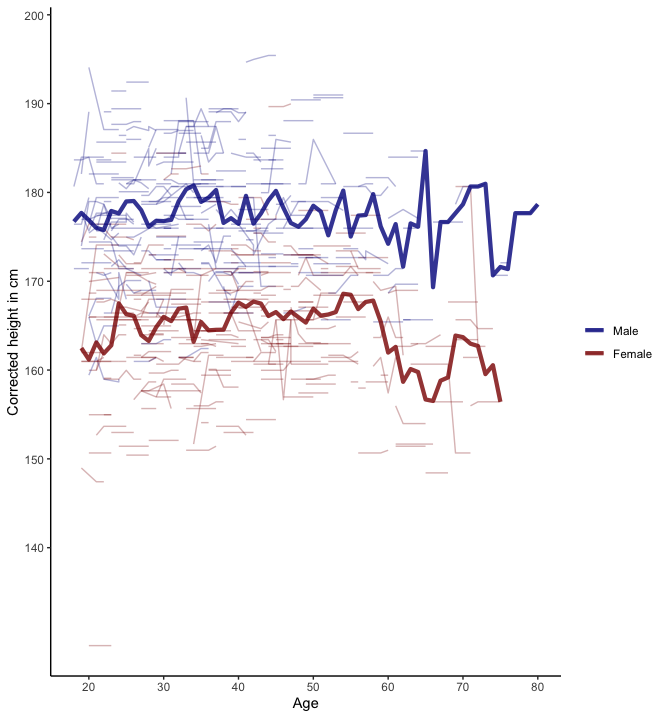
**

*Mean corrected height over time in women and men.*

**Supplementary Figure 3.** Longitudinal evolution of corrected BMI percentile in children

**
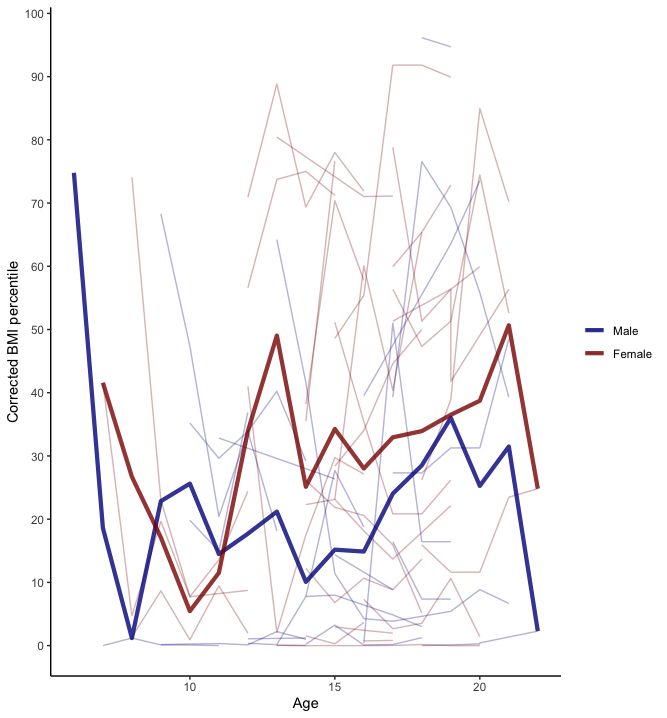
**

*Mean corrected BMI percentile in boys and girls.*

**Supplementary Table 4**: Comparison of growth mixture models with 2-5 classes representing differential BMI evolution trajectories

|  | **2 classes** | **3 classes** | **4 classes** | **5 classes** |
| --- | --- | --- | --- | --- |
| **Entropy** | 0.99 | 0.89 | 0.89 | 0.81 |
| **BIC (adjusted)** | 7622 | 7573 | 7543 | 7544 |
| **Lo Mendel-Rubin adjusted LRT** | 0.0005 | 0.08 | 0.31 | 0.41 |
| **Parametric bootstrapped likelihood ratio test** | 0.000 | < 0.001 | 0.000 | 0.19 |

*Growth mixture models with 2-5 latent classes. Abbreviations: BIC: Bayesian Information Criterion*

**Supplementary Figure 4.** Growth mixture models of subclasses with differential BMI evolution trajectories in adults


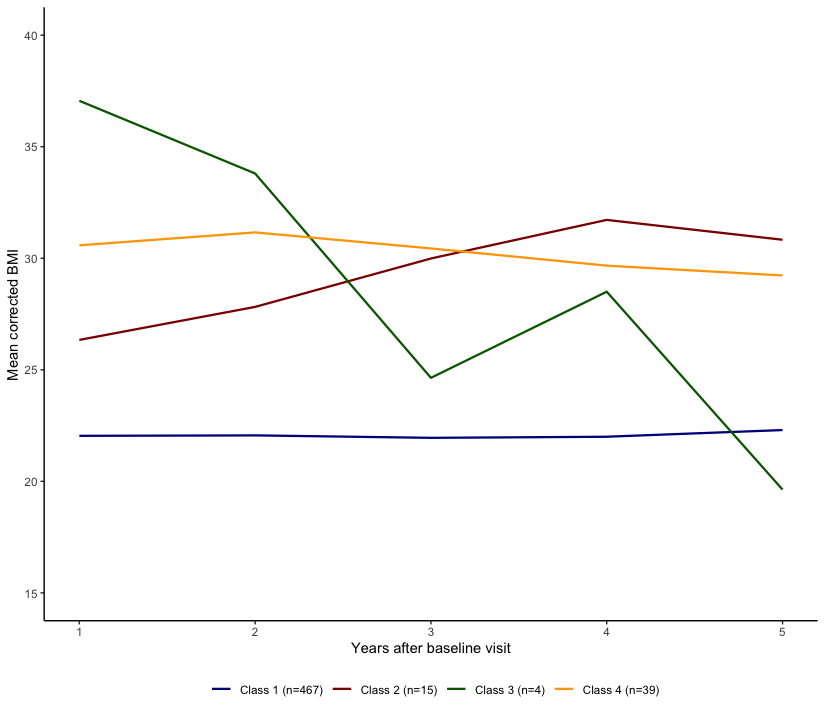


*Growth mixture model with 4 latent classes comprising a class with stable BMI evolution that remained within the normal range (n=467), a class with increasing BMI (n=15), a class with decreasing BMI (n=4) and a class with stable but obese BMI (n=39).*

**Supplementary Table 5**: Results of linear mixed effects model with SARA and ADL according to latent class

| **Analysis** | **Latent class** | **Estimate** | **Standard error** | **95% CIs** | **P-value** |
| --- | --- | --- | --- | --- | --- |
| SARA according to latent class | 2 | -1.4 | 2.3 | -6.0, 3.1 | 0.54 |
|  | 3 | 0.72 | 4.5 | -8.0, 9.5 | 0.87 |
|  | 4 | -3.1 | 1.5 | -6.1, -0.24 | 0.035 |
| ADL according to latent class | 2 | -1.0 | 1.9 | -4.8, 2.8 | 0.61 |
|  | 3 | 0.35 | 3.7 | -6.9, 7.6 | 0.92 |
|  | 4 | -1.3 | 1.2 | -3.7, 1-1 | 0.27 |

*Results of linear mixed models unadjusted for confounders with latent class 1 as the reference class. Adjusting for age and sex yielded similar results. Abbreviations: ADL: activities of daily living; SARA: scale for the assessment and rating of ataxia*

**Supplementary Table 6**: Demographic and clinical differences between latent classes at baseline

*
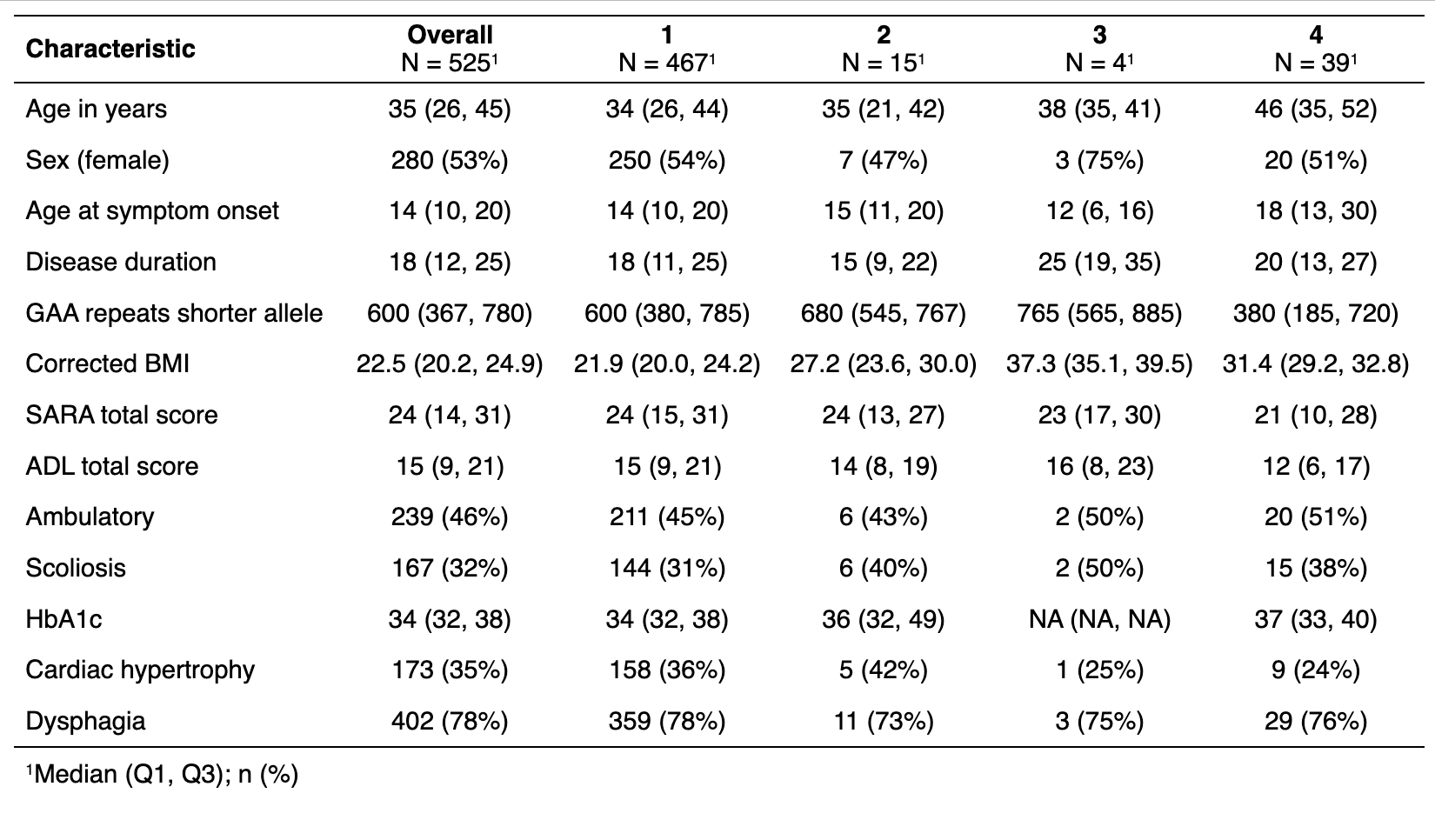
*

*Comparison of demographic and clinical characteristics between different latent classes. Abbreviations: ADL: activities of daily living; SARA: scale for the assessment and rating of ataxia. GAA: guanine adenine adenine*

**Supplementary Table 7**: Results of analyses unadjusted for confounders

| **Analysis** | **Estimate** | **Standard error** | **95% CIs** | **P-value** |
| --- | --- | --- | --- | --- |
| Overweight (BS) vs SARA score | -1.86 | 0.39 | -2.3, -1.4 | <0.001 |
| Underweight (BS) vs SARA score | -2.8 | 0.44 | -3.7, -1.9 | <0.001 |
| Overweight (BS) vs ADL score | -1.2 | 0.24 | -1.7, -0.68 | <0.001 |
| Underweight (BS) vs ADL score | -1.6 | 0.49 | -2.6, -0.5 | <0.001 |
| Overweight (BS) vs diabetes (HbA1c) | -1.36 | 4.3 | -9.8, 7.1 | 0.75 |
| Overweight (BS) vs cardiomyopathy | -0.63 | 0.49 | -1,5, 0.34 | 0.20 |

*Results of linear mixed models unadjusted for confounders. Normal weight represents the reference category. Abbreviations: BS: baseline; SARA: scale for the assessment and rating of ataxia*
